# Apelin promotes hepatic fibrosis through ERK signaling in LX-2 cells

**DOI:** 10.1007/s11010-019-03581-0

**Published:** 2019-07-03

**Authors:** Ying Wang, Jiayi Song, Hongyan Bian, Jiaqi Bo, Shuangyu Lv, Weitong Pan, Xinrui Lv

**Affiliations:** 1 The First Affiliated Hospital, Henan University, Kaifeng, 475004 Henan China; 2 The Key Laboratory of Receptors-Mediated Gene Regulation and Drug Discovery of School of Basic Medicine, Henan University, Kaifeng, 475004 Henan China; 3 Queen Mary School, Nanchang University, Nanchang, 330029 Jiangxi China

**Keywords:** Apelin, APJ, Liver fibrosis, NAFLD, LX-2 cells, ERK

## Abstract

Apelin participates in cardiovascular functions, metabolic disease, and homeostasis disorder. However, the biological function of apelin in liver diseases, especially liver fibrosis is still under investigation. The present study aimed to investigate the expression of apelin in nonalcoholic fatty liver disease (NAFLD) and the mechanism of apelin promoting hepatic fibrosis through ERK signaling in hepatic stellate LX-2 cells. The results showed that the ALT and AST levels in serum were increased in the mice fed HFC. The histological staining revealed that hepatocellular steatosis and ballooning degeneration was severe, and fibrogenesis appeared as increased pericellular collagen deposition along with pericentral (lobular) collagen deposition in the mice fed HFC. Immunochemistry and qRT-PCR results showed that the expression of apelin and profibrotic genes was higher as compared to the control group. The in vitro experiments demonstrated that apelin-13 upregulated the transcription and translation levels of collagen type I (collagen-I) and α-smooth muscle actin (α-SMA) in LX-2 cells. The immunofluorescent staining, qRT-PCR, and Western blot results showed that the overexpression of apelin markedly increased the expression of α-SMA and cyclinD1. The LX-2 cells treated with apelin-13 displayed an increased expression of pERK1/2 in a time-dependent manner, while the pretreatment with PD98059 abolished the apelin-induced expression of α-SMA and cyclinD1. Furthermore, the in vivo and in vitro assays suggested a key role of apelin in promoting liver fibrosis, and the underlying mechanism might be ascribed to the apelin expression of profibrotic genes via ERK signaling pathway.

**Electronic supplementary material:**

The online version of this article (10.1007/s11010-019-03581-0) contains supplementary material, which is available to authorized users.

## Introduction

Nonalcoholic fatty liver disease (NAFLD) is the most common form of chronic liver diseases in adults and children worldwide with a continual increase in the number of incidences [1]. Irrespective of the cause, liver diseases typically experience three major events, such as hepatic fibrosis, cirrhosis, and hepatocellular carcinoma. The severity of these events determined the prognosis and treatment of liver diseases. Previous studies confirmed that activated hepatic stellate cells (HSCs) are vital contributors to the fibrosis of the damaged liver [2, 3]. The activated HSCs are primarily responsible for the production of extracellular matrix (ECM) and the development of fibrosis in the liver. In the normal liver, quiescent HSCs (qHSCs) contain cytoplasmic lipid droplets primarily consisting of retinyl esters. Moreover, in response to injury or inflammatory stimulus, qHSCs are transformed to myofibroblast-like cells, which highly express the α-smooth muscle actin (α-SMA) and collagen type I (collagen-I) [4, 5]. Consequently, the activated HSCs exhibited high proliferation and migration in the liver injury sites.

Apelin is an endogenous ligand for the APJ receptor (angiotensin II receptor-like-1, AT-1). The prepro-apelin consisted of 77 amino acids that could be cleaved into several biologically active forms of peptides, such as apelin-13, -16, -17, and -36. Each isoform harbored a potent activator for APJ. Both apelin-13 and apelin-17 exerted a stronger activity than apelin-36 [6–8]. Furthermore, apelin played pivotal and various roles in the physiological and pathophysiological processes, including regulation of blood pressure, cardiac contractility, angiogenesis, metabolic balance, cell proliferation, apoptosis, and inflammation [9–12]. In addition, it has been shown that in the liver, apelin participated in hepatocyte apoptosis, glycogen synthesis, and fibrosis formation [13, 14]. A recent clinical investigation reported that serum apelin was associated with the histological and hemodynamic states of chronic liver disease [15]. In addition, previous studies demonstrated that hypothalamic apelin regulated the hepatic glucose metabolism in mice fed a high-fat diet [16], and high levels of serum apelin-12 were observed in human NAFLD [17]. These clinical studies suggested that apelin was positively correlated with the homeostasis model assessment (HOMA) index and body mass index (BMI) in metabolic liver disease. Also, the expression of apelin was increased sharply in the hepatic tissue of cirrhotic rats or humans [4]. Interestingly, apelin was highly expressed under hypoxic or proinflammatory conditions in HSCs, and it might promote liver fibrosis or cirrhosis progression [18, 19]. Some studies demonstrated that apelin bound to APJ led to the phosphorylation of extracellular signal-regulated kinase (ERK), protein kinase B (Akt), and p70S6 kinase signaling pathways in cell proliferation or migration activities [20–22]. However, whether the apelin could promote liver fibrosis by ERK signaling pathway remains elusive.

In the present study, we aimed to investigate the function and mechanism of apelin in promoting hepatic fibrosis in LX-2 cells and the mice fed high-fat chow (HFC).

## Materials and methods

### Animal experiments

C57BL/6 mice (SPF, males, 6–8-week-old, 22–24 g) were purchased from the Institutional Animal Care and Use Committee of Charles River (Beijing, China, License number: SCXK 2012-0001). All procedures performed in studies involving animals were carried out in accordance with the United States NIH guidelines (Guide for the Care and Use of Laboratory Animals (1985), DHEW Publication No. (NIH) 85–23: Office of Science and Health Reports, DRR/NIH, Bethesda, MD, USA) and the ethical standards of Animal Research Ethics Committee of Henan University (The experimental number: HUSOM-2017-200). The mice were divided into two groups (n = 12), the model group was fed HFC that contained 45% fat (MD12032, Medicine Led, Yangzhou, China) for 24 weeks, while the normal mice were fed a diet containing 10% fat (MD12031, Medicine Led) for the same length of time. The diet consumption and body weight were recorded weekly. At the end of 24 weeks of feeding, the mice were sacrificed, blood was collected and centrifuged, and the serum was stored at − 80 °C for biochemical analyses. The livers were harvested, snap frozen for further analysis, such as PCR and Western blotting, or stored in 10% formalin for histopathology staining.

### Cell culture and treatment

LX-2 cell line was purchased from Meixuan Biological Science and Technology Ltd (Shanghai, China). The cells were seeded in 60-mm plates to confluency for 24 h in high glucose DMEM (Dulbecco’s modified Eagle’s medium) supplemented with 10% FBS (fetal calf serum) in a humidified atmosphere with 5% CO_2_ at 37 °C. Subsequently, LX-2 cells were cultured in serum-free DMEM for 24 h, followed by treatment with apelin-13 (100 nM) (Sigma–Aldrich) for the indicated time points.

### Hematoxylin–eosin (H&E) staining

The liver tissues obtained from the mice were fixed with 10% neutral-buffered formalin at room temperature for 24 h, embedded in paraffin, sectioned to a thickness of approximately 5 μm, and stained with H&E according to the standard protocol and examined by light microscopy.

### Sirius red staining

The liver tissue sections were deparaffinized and hydrated in distilled water. Picrosirius red solution was applied to cover the tissue section and incubated for 60 min. Subsequently, the slides were rinsed with an acetic acid solution, followed by dehydration with absolute alcohol. Finally, the slide was mounted in synthetic resin.

### Oil red-O staining

Frozen liver tissues were cut into 7 mm, then, fixed with 10% (v/v) formaldehyde for 15 min at room temperature. After washing three times with double-distilled water (ddH_2_O), the slides were incubated with filtered Oil Red O solution at room temperature for another 15 min, followed by further washing five times with ddH_2_O to remove the background staining. The liver tissue sections were observed and photographed randomly under the microscope.

### Biochemical analyses

The blood of mice was collected in a common tube from an angular vein after the animals were executed, and allowed to stand for 4–6 h at room temperature, followed by centrifugation at 3000×*g* for 5 min. The serum was collected, and serum alanine aminotransferase (ALT), aspartate aminotransferase (AST), and cholesterol levels were determined by Reitman Frankel method using commercially available kits (Jiancheng Bioengineering Institute, Nanjing, China).

### Immunohistochemistry (IHC)

The liver tissues of mice were fixed with 10% neutral-buffered formalin at room temperature for 24 h, embedded in paraffin, sectioned to a thickness of approximately 5 μm, and immunostained by a standard protocol. The immunostaining of the sections was performed using anti-apelin (GeneTex), anti-APJ (Abcam), anti-α-SMA (Abcam), and anti-cyclinD1 (Abcam) antibodies (1:100) and counterstained with hematoxylin. The staining intensities were determined by measuring the IOD (integrated optical density) with light microscopy using the computer-based Image-Pro Morphometric System in a double-blind manner.

### RNA preparation and qRT-PCR (quantitative reverse transcription-PCR)

Total RNA was isolated with TRIzol^®^ reagent (Invitrogen) according to the manufacturer’s instructions. RNA concentrations and purity were determined by measuring the absorbance A260–A280 nm ratio. As an internal control, GAPDH (glyceraldehyde-3-phosphate dehydrogenase) gene primers were used for RNA template normalization. Quantitative PCR of the related genes was performed using a Platinum SYBR Green qPCR SuperMix UDG Kit (Invitrogen). The following primers were used: apelin (mouse), 5′TCTTGGCTCTTCCCTCTTTTCA 3′ (sense) and 5 5′GTGCTGGAATCCACTGGAGAA 3′ (antisense); APJ (mouse), 5′TCGGCTAAGGCTGCGAGTC 3′ (sense) and 5′CGTCTGTGGAACGGAACAC 3′ (antisense); α-SMA (mouse), 5′GTCCCAGACATCAGGGAGTAA 3′ (sense) and 5′TCGGATACTTCAGCGTCAGGA 3′ (antisense); collagen-I (mouse), 5′ GCTCCTCTTAGGGGCCACT3′ and 5′ CCACGTCTCACCATTGGGG3′; TGF-β1(mouse), 5′ CTCCCGTGGCTTCTAGTGC3′(sense) and 5′GCCTTAGTTTGGACAGGATCTG 3′(antisense); IL-10(mouse), 5′ TAACTGCACCCACTTCCCAG3′(sense) and 5′AGGCTTGGCAACCCAAGTAA 3′ (antisense); GAPDH (mouse), 5′TGTGAACGGATTTGGCCGTA3′ (sense) and 5′ACTGTGCCGTTGAATTTGCC3′ (antisense); apelin (human) 5′, GCTCTGGCTCTCCTTGACC3′ (sense) and 5′CCATTCCTTGACCCTCTGG 3′ 3′(antisense); collagen-I (human), 5′ GAGGGCCAAGACGAAGACATC3′ (sense) and 5′ CAGATCACGTCATCGCACAAC3′ (antisense); α-SMA (human), 5′GTGTTGCCCCTGAAGAGCAT 3′ (sense) and 5′GTGTTGCCCCTGAAGAGCAT 3′ (antisense); GAPDH (human), 5′GGAGCGAGATCCCTCCAAAAT3′ (sense) and 5′GGCTGTTGTCATACTTCTCATGG3′ (antisense); The relative expression level was calculated using the following equation:relative gene expression = 2^−∆∆CT^.

### Western blot

Crude proteins were extracted from liver tissues of mice or LX-2 cells as described previously [23, 24], resolved by SDS/PAGE and transferred on to a PVDF membrane (Millipore). Membranes were blocked with 5% (w/v) non-fat dried skimmed milk powder in TTBS (100 mM Tris/HCl, pH 7.5, 150 mM NaCl and 0.5% Tween 20) for 2 h at 37 °C and then incubated overnight at 4 °C with the following primary antibodies: 1:300 dilution rabbit anti-apelin (GeneTex), 1:1000 dilution rabbit anti-α-SMA (Abcam), 1:2500 dilution rabbit anti-cyclinD1 (Abcam), anti-β-actin and rabbit anti-IgG (Santa Cruz Biotechnology) antibodies. After incubation with the appropriate secondary antibody, the immunoreactive signal of antibody-antigens were visualized using the Chemiluminescence plus Western blot analysis kit (Tanon Biotechnology).

### Immunofluorescent staining

LX-2 cells were fixed in 4% paraformaldehyde and permeabilized with 0.1% Triton X-100, incubated with anti-α-SMA antibody, and further stained with Cy3-conjugated secondary antibody. Staining of 4′,6-diamidino-2-phenylindole (DAPI) was used to visualize nuclear localization. Each sections was observed under an inverted fluorescence microscope (Leica).

### Plasmids transfection

Human apelin expression plasmids were purchased from Origene Technologies Inc. The transfection was performed using Lipofectamine™ reagent (Invitrogen) following the manufacturer’s instructions. At 24 h following transfection, LX-2 cells were treated with or without apelin (100 nM). Then, the cells were harvested and used for RT-PCR and Western blot.

### SiRNA transfection

Small interfering RNA (siRNA)-targeting human apelin (si-apelin) and nonspecific siRNA (si-Con) were purchased from Santa Cruz Biotechnology. Transfection was performed using a Lipofectamine reagent (Invitrogen) according to the manufacturer’s instructions. At 24 h posttransfection, LX-2 cells were harvested and used for qRT-PCR and Western blot assays.

### The detection of ERK signaling

LX-2 cells were treated with 100 nM apelin-13 for the indicated time points. Total cell lysates were analyzed by Western blot using antibodies against p-ERK and ERK. LX-2 cells were pretreated with PD98059 (25 mmol/L) for 2 h, followed by a 24 h incubation with or without apelin-13 (100 nM). Subsequently, the cells were collected, and the expression of α-SMA and cyclinD1 proteins was examined by Western blot using the respective antibodies.

### Statistical analyses

Data are presented as histograms of mean ± SD (standard deviation) from three or more independent experiments. Statistical analyses were performed using the Student’s *t* test or one-way ANOVA and post hoc test according to the number of groups compared. *P *< 0.05 indicated statistically significant differences.

## Result

### Morphological staining of the liver tissues and the biochemical analysis in NAFLD of the mice

**Fig. 1:**
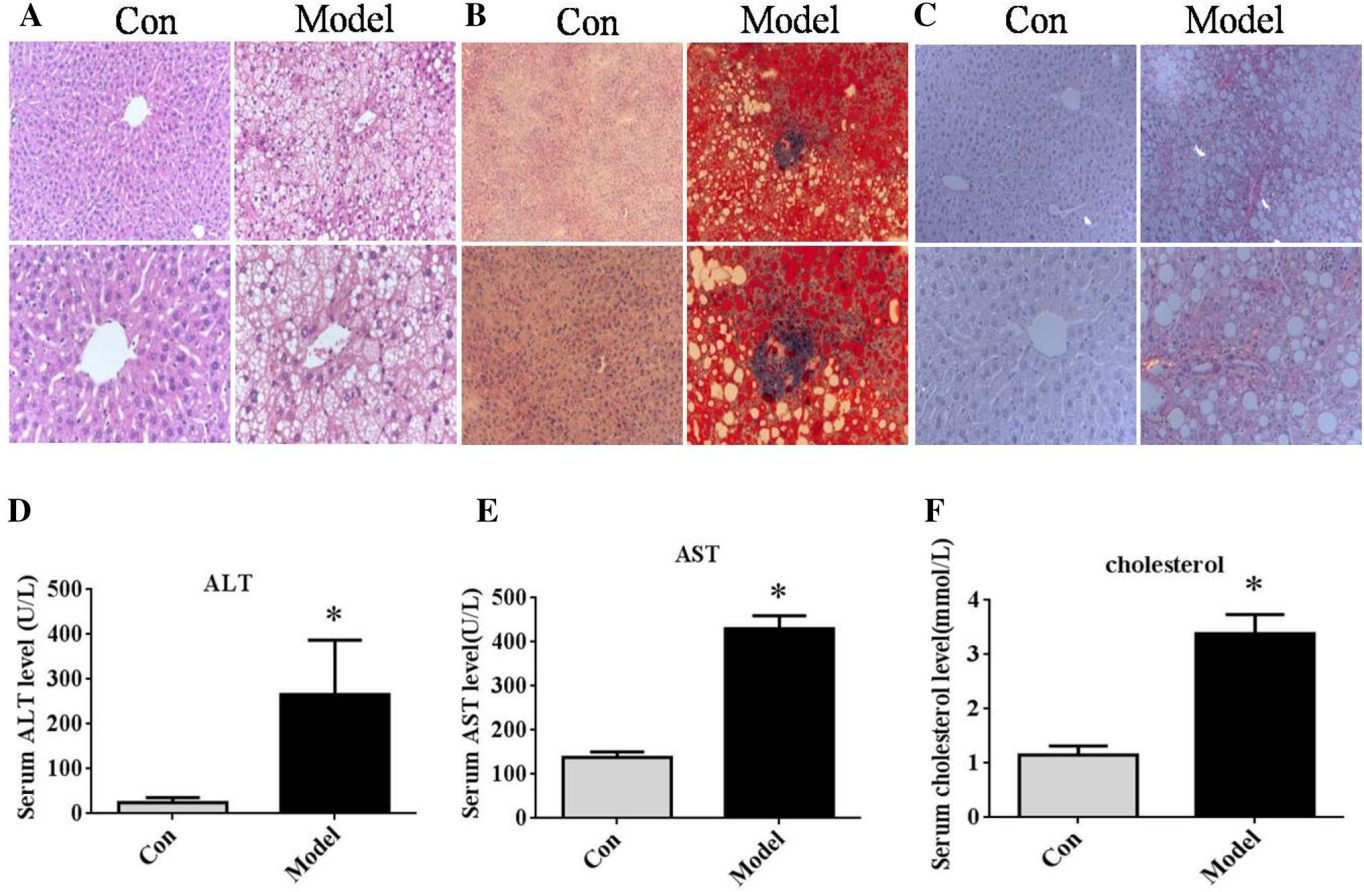
The morphological staining of the liver tissues and the biochemical analysis in the NAFLD of mice. **a** Representative images of liver histology (H&E staining, magnification: upper × 200, lower × 400), **b** Representative photomicrographs of liver oil red staining (magnification: upper × 200, lower × 400), **c** Representative photomicrographs of liver Sirus red staining (magnification: upper × 200, lower × 400), **d**, **e**. Serum ALT and AST levels. *P < 0.01 compared to the control group. F. Serum cholesterol levels. *P < 0.01 compared to the control group (n = 12 in each group)

In this study, liver fibrosis was induced in mice through continuous feeding of HFC for 24 weeks. H&E staining of the liver sections from the model mice revealed excessive steatosis and ballooning degeneration in the disorganized hepatocytes, as well as the disordered hepatic lobule structure in the surrounding portal vein (Fig. 1a). Oil red staining showed lipid accumulation and that droplets accumulated remarkably in the liver tissues of the model mice as compared to the control group (Fig. 1b). Fibrosis appeared as increased pericellular collagen deposition along with pericentral (lobular) collagen deposition in Sirius red-stained sections of model mice liver tissues (Fig. 1c). In addition, biochemical analysis showed that serum ALT, AST, and cholesterol levels were significantly increased in the mice fed HFC as compared to the controls (Fig. 1d–f). Taken together, these data indicated that the structure of the liver was damaged and the function of hepatocytes disturbed in NAFLD mice due to feeding HFC.

### Expression of apelin, APJ, and the related profibrotic genes in NAFLD of the mice

**Fig. 2:**
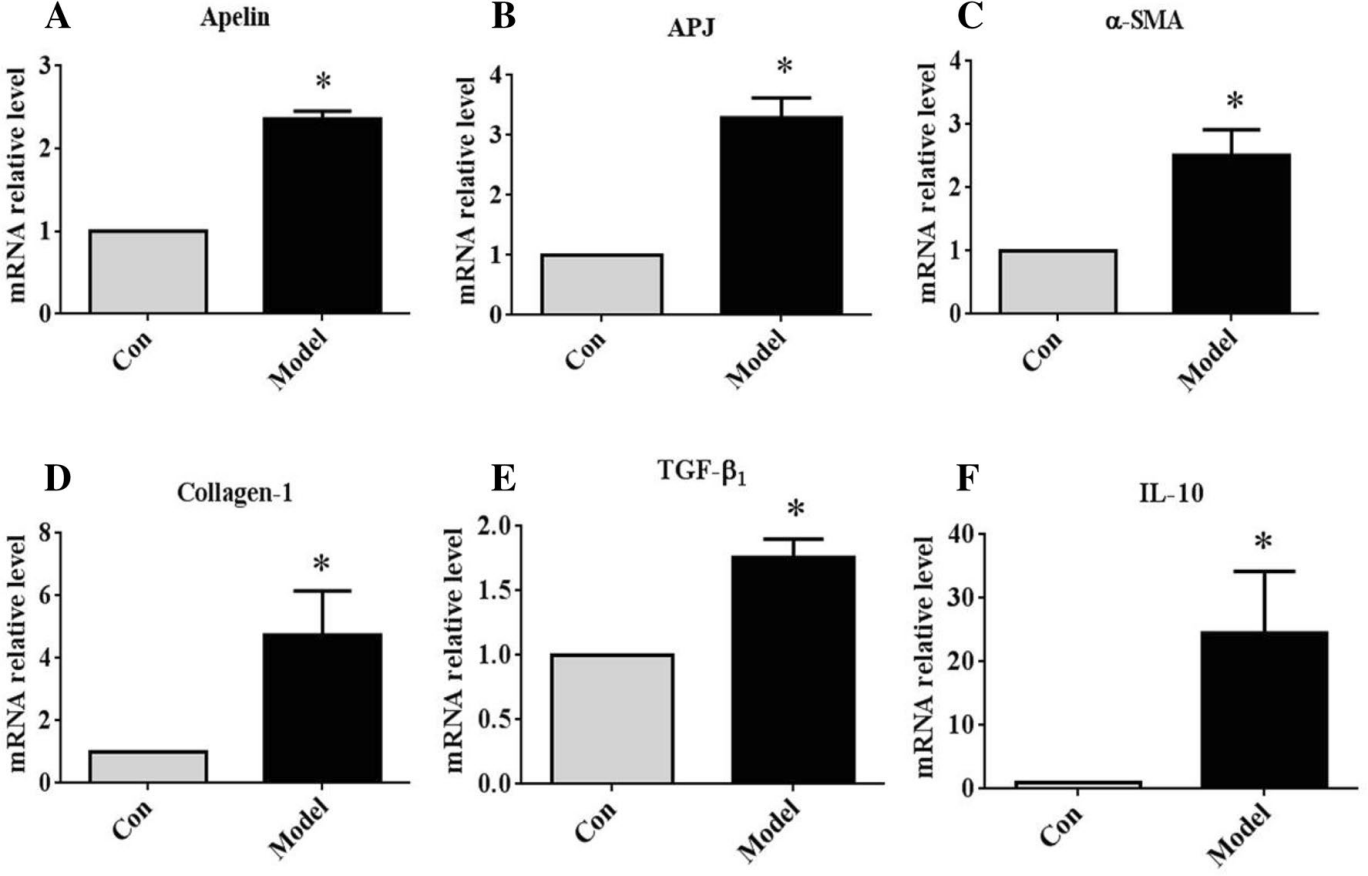
The expression of apelin, APJ, and the related profibrosis genes of the liver tissues in the NAFLD of mice. **a**–**f** Total RNA was transcribed with reverse transcriptase and amplified by PCR. GAPDH was used as an internal control. The mRNA expression of apelin, APJ, α-SMA, collagen-1, TGF-β, and IL-10 was examined by qRT-PCR from the liver tissues of the mice. *P < 0.01 compared to the control group (n = 12 in each group)

**Fig. 3:**
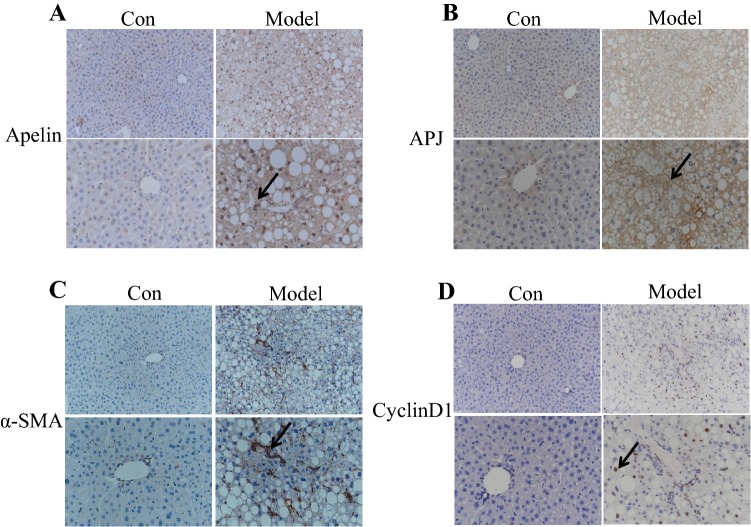
The expression of apelin, APJ, α-SMA, and cyclinD1 in the IHC staining of the liver tissues in the NAFLD of mice. **a**–**d** IHC staining of apelin, APJ, α-SMA, and cyclinD1 protein in the liver tissues of the mice (n = 12 in each group; magnification: upper × 200, lower × 400). The arrow point to specific proteins

The mRNA levels of apelin, APJ, and the profibrosis-related genes, such as α-SMA, collagen-I, TGF-β, and IL-10, (a marked proinflammatory factor) were upregulated in the model group as compared to the control mice (Fig. 2a–f). In addition, the IHC staining revealed the expression of apelin, APJ, α-SMA, and cyclinD1 in the mice of the model group as assessed by qRT-PCR, and the arrow point to specific proteins (Fig. 3a–d). These results indicated that apelin and APJ participated in the hepatic steatosis in NAFLD of mice.

### Apelin-13 increased the α-SMA and collagen-I mRNA and protein levels in LX-2 cells

**Fig. 4:**
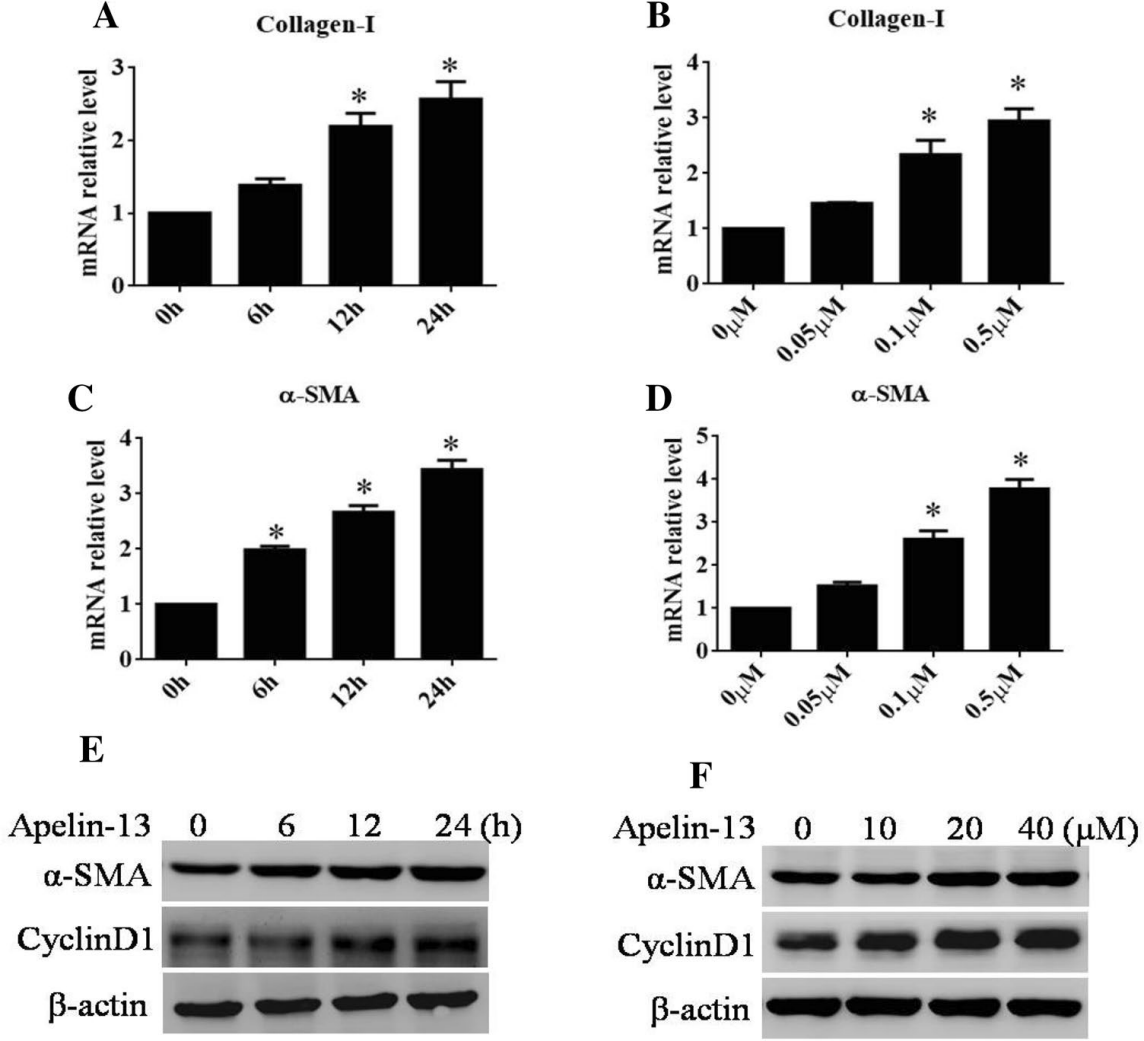
Apelin-13 promoted α-SMA and collagen-I mRNA and protein levels in LX-2 cells. LX-2 cells were treated with apelin-13 (100 nM) for various time points or at different doses (for 24 h). **a**–**d** Total RNA was transcribed with reverse transcriptase and amplified by PCR. GAPDH was used as an internal control. *P < 0.01 compared to the control group. **e**, **f** Western blot was performed using anti-α-SMA and anti-cyclinD1 antibodies to examine the expression of α-SMA and cyclinD1 at various time points or different doses. β-actin was used as the endogenous control

LX-2 cells belong to the hepatic stellate cell (HSC) line, and activated HSCs play a critical role in liver fibrosis. To assess whether the expression of the related marked genes, α-SMA, and collagen-I, was upregulated in apelin-stimulated LX-2 cells, RT-PCR and Western blotting methods were employed to examine the expression with respect to transcription and translation as a response to apelin signaling. When LX-2 cells were treated with 100 nM apelin-13 for various time points, the level of α-SMA and collagen-I mRNA increased in a time- and dose-dependent manner (Fig. 4a–d). In addition, the paraplastic hepatocyte was one of the primary causes of liver fibrosis, and the Western blot results showed that the expression level of cyclinD1, one of the cell cycle marker proteins, apparently enhanced the apelin-13 signaling pathway (Fig. 4e, f). These results demonstrated that apelin participated in the formation of liver fibrosis in LX-2 cells.

### Apelin played a positive regulatory role in profibrotic genes expression

**Fig. 5:**
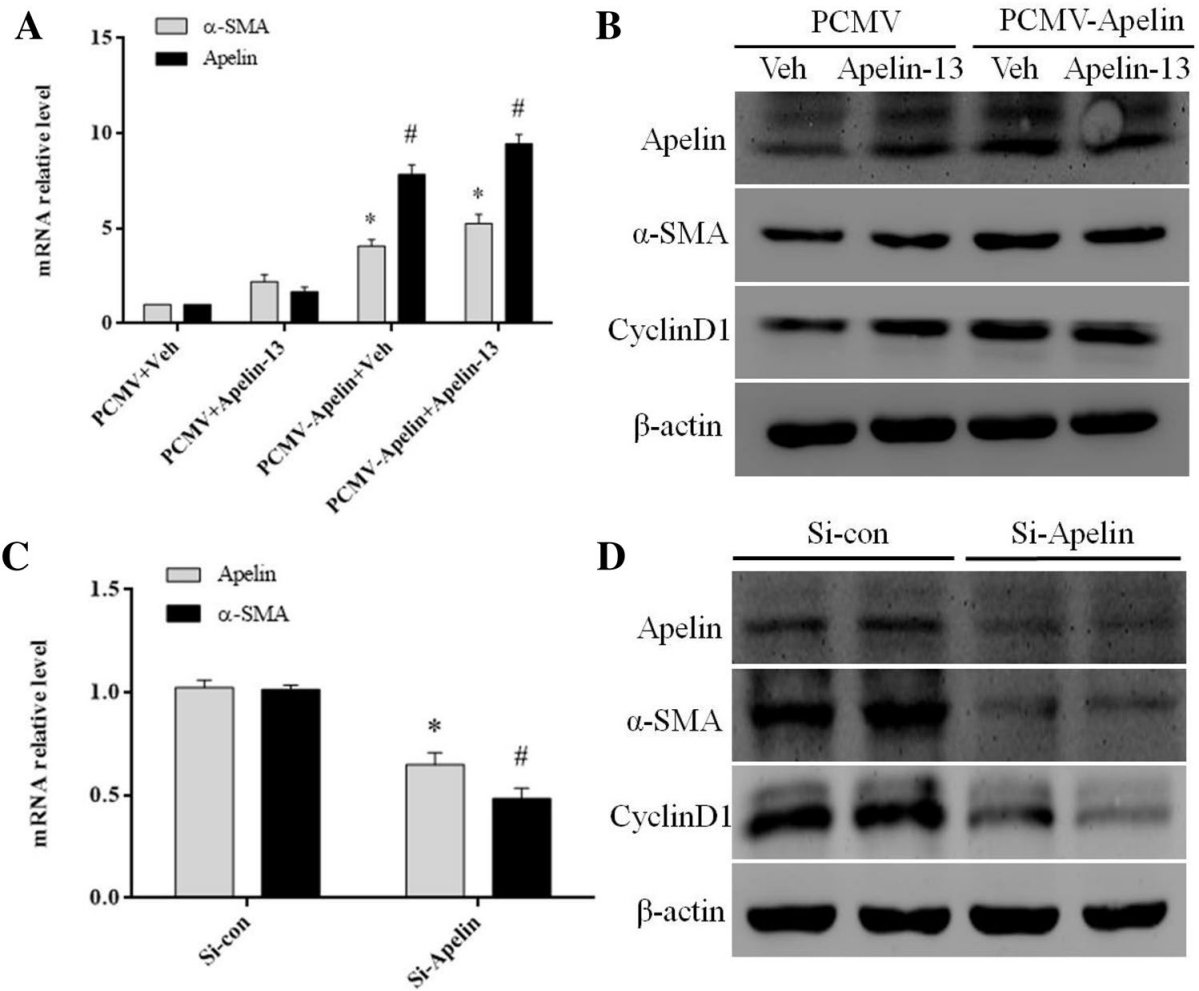
Overexpression or knockdown of apelin regulated α-SMA mRNA and protein levels in LX-2 cells. **a**, **b** Apelin expression plasmids were transfected via Lipofectamine™ reagent following the manufacturer’s instructions. At 24 h after transfection, LX-2 cells were treated with or without apelin-13 (100 nM). **c**, **d** LX-2 cells were transfected si-apelin or si-con for 24 h, Then, the cells were harvested and used for qRT-PCR and Western blot. Total RNA was transcribed using reverse transcriptase and amplified by PCR. GAPDH was used as an internal control. *P < 0.05 compared to the control group. Western blot was performed using anti-α-SMA and anti-cyclinD1 antibodies to examine the protein expression. β-actin was used as the loading control

**Fig. 6:**
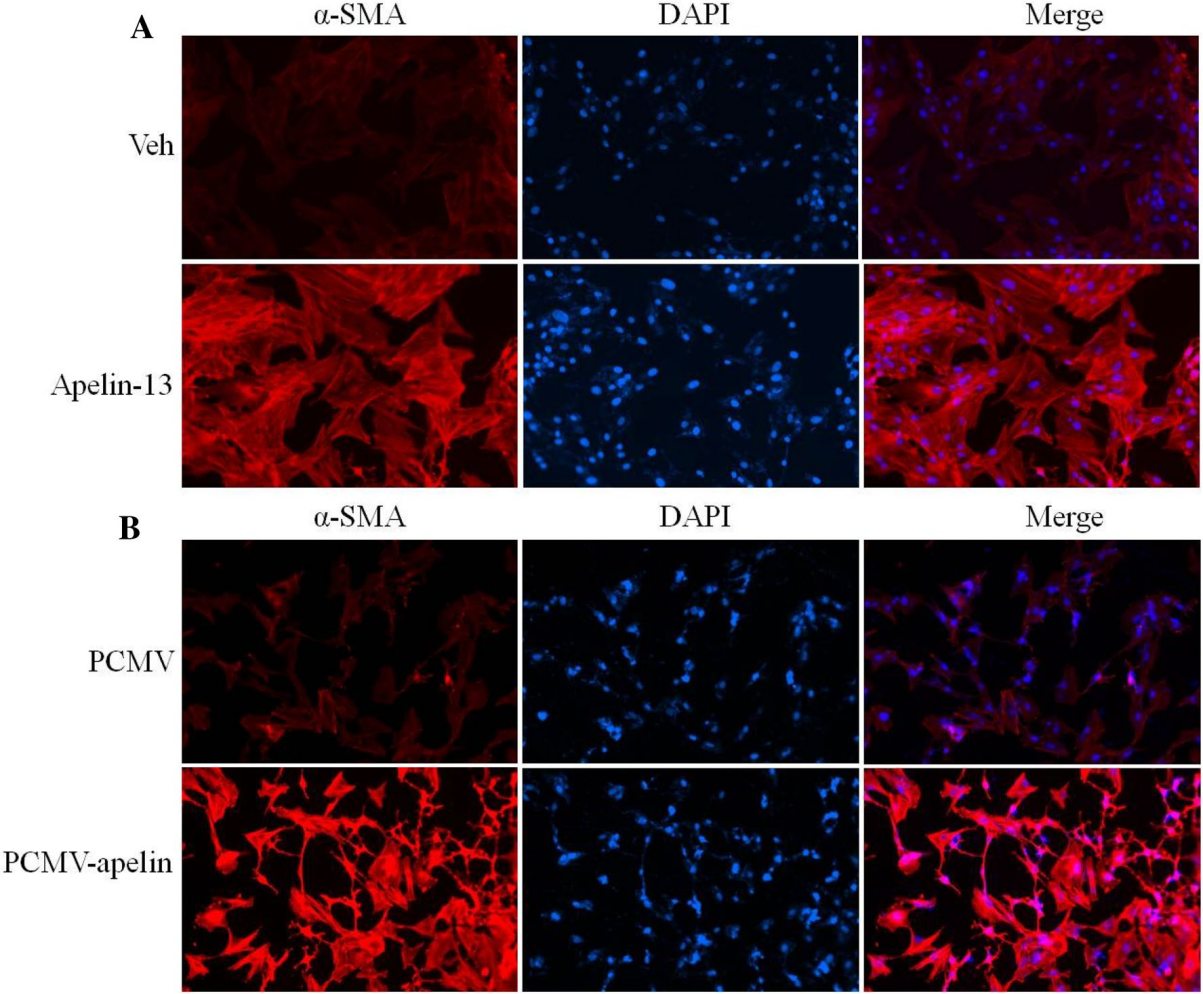
The effects of apelin on immunofluorescent staining of LX-2 cells. **a** LX-2 cells were treated with apelin-13 (100 nM) or **b** LX-2 cells were transfected si-apelin for 24 h, then followed by fixation in 4% paraformaldehyde, permeabilization with 0.1% Triton X-100, incubation with anti-α-SMA primary antibody, and further staining with the Cy3-conjugated secondary antibody. DAPI staining was utilized to visualize the nuclear localization. Each section was observed under an inverted fluorescence microscope

To further verify the role of apelin in regulating the expression of fibrogenesis-related genes, apelin expression plasmids were transfected in LX-2 cells to overexpress apelin. The RT-PCR and Western blot results showed that the overexpression of apelin markedly increased the expression of α-SMA and cyclinD1 at the transcription and translation levels (Fig. 5a, b). Conversely, knockdown endogenous apelin expression with si-apelin, the results showed that the expression of α-SMA and cyclinD1 markedly decreased at both mRNA and protein levels (Fig. 5c, d). Additionally, the immunofluorescent staining showed that when LX-2 cells were stimulated for 24 h by apelin-13, the α-SMA expression was distinctly upregulated (Fig. 6a). Another, the number of α-SMA (red)-positive cells was more and the expression of α-SMA was greatly increased in apelin-overexpressed LX-2 cells as compared to that of the control from the immunofluorescence results (Fig. 6b). These results suggested that apelin positively regulated the α-SMA expression in LX-2 cells.

### Apelin-promoted α-SMA expression through ERK signaling in LX-2 cells

**Fig. 7:**
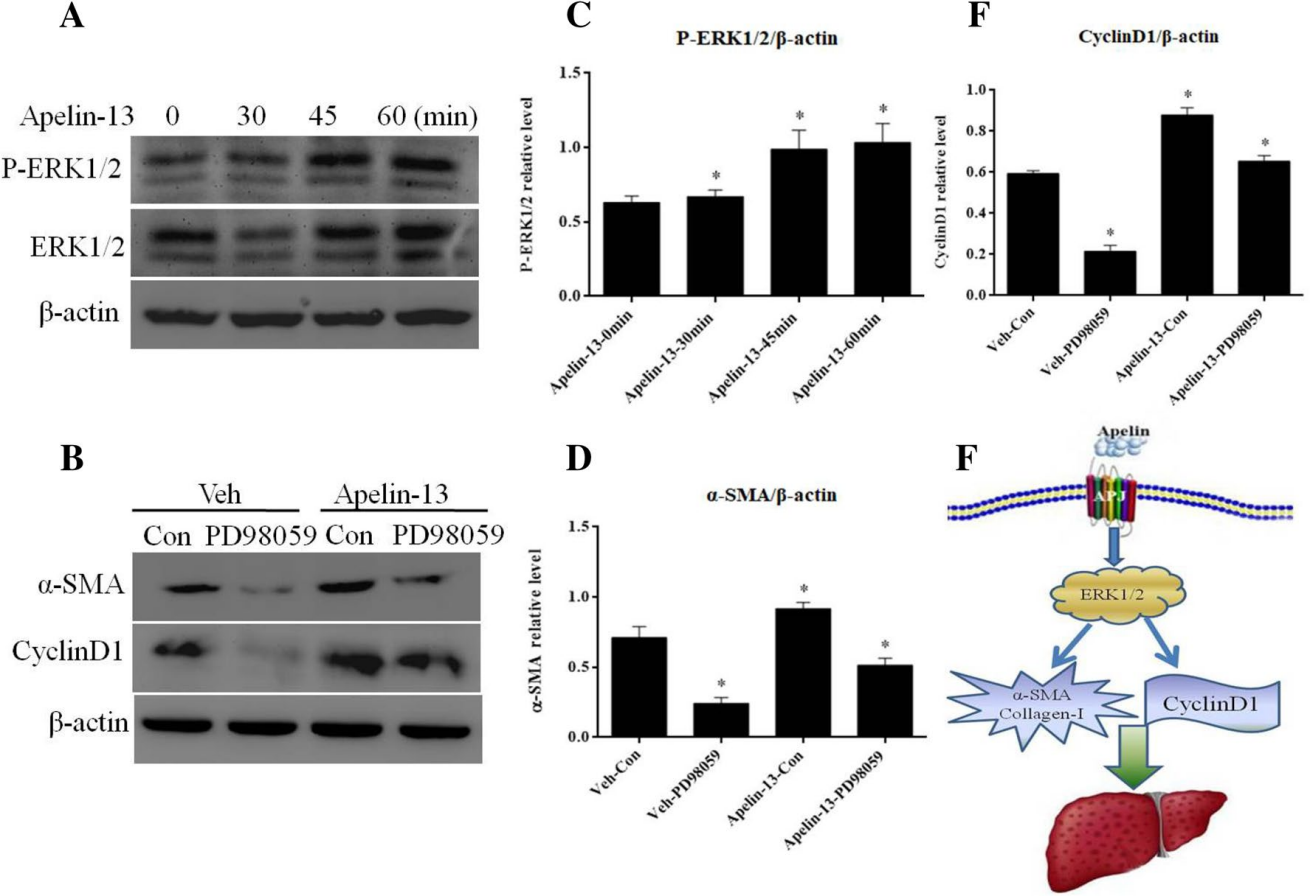
Apelin promoted the expression of α-SMA through ERK signaling in LX-2 cells. **a** LX-2 cells were treated with 100 nM apelin-13 for the indicated time points. Total cell lysates were analyzed by Western blot using antibodies against p-ERK and ERK. β-actin was used as the endogenous control. **b** LX-2 cells were pretreated with PD98059 (25 mmol/L) for 2 h, followed by a 24 h incubation with or without apelin-13 (100 nM). Subsequently, the cells were collected, and the expression of α-SMA and cyclinD1 proteins was examined by Western blot using the respective antibodies. β-actin was used the endogenous control. **c**–**e** The densitometry analysis of the band intensities was quantized by Photoshop Image Tanon software. *P < 0.05 versus control group. **f** The pattern graph of apelin promotion the expression of α-SMA through ERK signaling

The effect of apelin-13 on the expression of ERK1/2 and pERK1/2 was demonstrated by Western blot analysis. Figure 7a, c showed that the level of pERK1/2 was increased within 45 min and persisted up to 1 h after apelin-13 treatment. To further verify the role of ERK signaling in regulating the expression of profibrotic genes, LX-2 cells were pretreated with the ERK inhibitor, PD98059, for 2 h before exposure to apelin-13. Consequently, the pharmacological inhibition of ERK blocked the apelin-induced increase in α-SMA and cyclinD1 levels (Fig. 7b, d, e). These results suggested that ERK signaling pathway mediated the apelin-induced expression of profibrogenic genes in LX-2 cells (Fig. 7f).

## Discussion

NAFLD pathogenesis is associated with inflammation, steatosis, and fibrosis of chronic liver injury [25–27]. The present study established the genetically unaltered C57BL/6J mice model on HFC for 24 weeks. The results showed that ballooning degeneration and steatosis, as well as, lipid accumulation and fibrosis made a remarkable appearance in the liver tissues of the model mice (Fig. 1a). Furthermore, the mRNA expression of profibrotic-related genes, α-SMA, collagen-I, TGF-β, and IL-10 (Fig. 2c–f), was markedly increased in this mice model, and the results were consistent with the pathogenesis of NAFLD. Previous studies had confirmed a series different of cell signaling pathways in the pathogenesis of NAFLD [28–31]. However, the etiology of NAFLD is yet elusive. The present study demonstrated that the expression of apelin/APJ was increased in the mice fed HFC, and apelin-13 promoted hepatic fibrosis by ERK signaling in LX-2 cells.

It had been demonstrated that apelin played a vital role in the pathophysiology of many diseases, including cardiovascular disease, renal disease, type 2 diabetes, and tumors [11, 32–35]. Apelin is highly expressed in the lung, heart, mammary glands, brain, kidney, testes, and ovaries [7, 36]; however, the expression was low in normal liver [20]. A previous study reported that the apelin expression was increased sharply in the hepatic tissue of cirrhotic rats than that in the controls [4]. Furthermore, the level of circulating apelin was markedly increased in rats with cirrhosis than that in the controls. In the APJ^−/−^ mice, the liver apoptosis and injury was significantly alleviated as compared to that in the wild-type mice [37]. The current results demonstrated that the expression of apelin and APJ was distinctly elevated in the liver tissues of the mice fed HFC than that in the control mice (Fig. 2a, b). Some studies reported that the level of serum apelin was increased in some liver diseases, such as NAFLD and cirrhosis [15–17]. These results suggested that apelin participated in the hepatic steatosis or fibrosis in NAFLD.

Interestingly, liver fibrosis was characterized by the excessive deposition of fibrillar collagen, especially collagen I, collagen III, and α-SMA. Therefore, the accumulation of activated HSCs was inhibited by modulating their stimulation or proliferation or promoting apoptosis, which is the leading target in preventing hepatic fibrosis [38]. The in vitro study demonstrated that apelin-13 increased the α-SMA and collagen-I mRNA and protein levels in LX-2 cells in a time- and dose-dependent manner (Fig. 4a–d). The immunofluorescent staining revealed an increased expression of α-SMA in apelin-13 stimulated LX-2 cells (Fig. 6a). The paraplastic hepatocyte is one of the main causes of liver fibrosis; the expression of cyclinD1 was increased in the apelin-13-treated LX-2 cells (Fig. 4e, f). The results demonstrated that the overexpression or knockdown of apelin markedly increased or decreased the expression of α-SMA and cyclinD1 at the transcriptional and translation levels in LX-2 cells (Fig. 5a–d), thereby rendering its role in promoting the proliferation and inducing fibrogenesis in the LX-2 cells. This phenomenon was consistent with that of the previous studies, where in apelin exerted a key role in regulating cell proliferation and apoptosis. Some studies also confirmed that the expression of apelin and APJ was low in the liver tissues of normal rats or human as compared to that in the case of cirrhosis [4]. The study demonstrated that AngII and ET-1 might exert some of their profibrotic effects in cirrhosis by activating the apelin signaling pathway [19]. However, the mechanism underlying the apelin-promoted liver profibrogenesis is yet unknown. The studies also confirmed that apelin interacted with APJ to promote the phosphorylation of p70S6K, and in turn, stimulated two signaling cascades—ERK and PI3K pathways [39, 40]. To gain further insight into the molecular mechanisms underlying the apelin-induced profibrogenesis and proliferation, as well as the α-SMA and cyclinD1 expression in LX-2 cells, we examined the intracellular signaling pathways. In the present study, we found that apelin-13 increased the expression of pERK1/2 in 45 min that persisted up to 1 h in LX-2 cells. In addition, PD98059, a pharmacological inhibitor of ERK, blocked the apelin-induced increase in α-SMA and cyclinD1 levels in LX-2 cells (Fig. 7a–e). These results suggested that ERK signaling pathway mediated the expression of apelin-induced profibrogenic and proliferation genes in LX-2 cells.

In summary, the current results showed that the expression of apelin/APJ was obviously increased in the mice fed HFC. Furthermore, apelin induced the profibrogenic and proliferation via ERK signaling pathways in LX-2 cells. Therefore, this study extended the knowledge in the field regarding the role of apelin in the development of liver fibrosis.

## Electronic supplementary material

Below is the link to the electronic supplementary material.

Supplementary material 1 (DOCX 54 kb)
