# Supplementary material for: Apelin promotes hepatic fibrosis through ERK signaling in LX-2 cells
Source: Mol Cell Biochem. 2019 Jul 3;460(1):205–15. doi: 10.1007/s11010-019-03581-0 (PMC6745032; doi:10.1007/s11010-019-03581-0)
Supplement: Supplementary file 1 — Supplementary material 1 (DOCX 54 kb) [file 11010_2019_3581_MOESM1_ESM.docx]

**Apelin promotes hepatic fibrosis through ERK signaling in LX-2 cells**

Yin Wang^1,2^, Jiayi Song^2^, Hongyan Bian^2^, Jiaqi Bo^2^, Shuangyu Lv^2^, Weitong Pan^3^, Weidong Chen^2^*, Xinrui Lv^2^*

^1^The First Affiliated Hospital, ^2^The Key Laboratory of Receptors-Mediated Gene Regulation and Drug Discovery of School of Basic Medicine, Henan University, Kaifeng, Henan, 475004, China, ^3^Jiangxi Medical College, Nanchang University＆Queen Mary University

* Correspondence:Email: lvxinrui@126.com

**Cell proliferation assay**


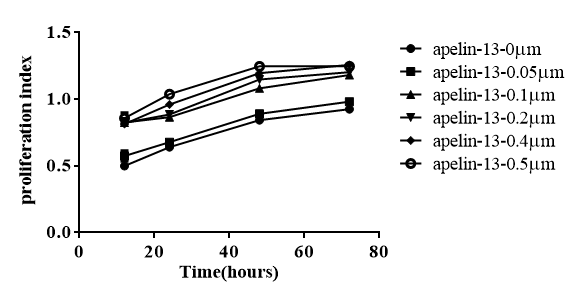
LX-2 cells were seeded into 96-well plates at a density of 2×10^3^ cells/well. After incubating for 24 h, cells were treated with apelin-13 (0–0.5 µM) for 12, 24, 48 and 72 h. Three parallel wells were set for each concentration. Twenty-four hours later, 10 µL of MTT (Sigma-Aldrich, St. Louis, MO, USA) at 5 mg/mL was added to each well for an incubation of 4 h. The supernatant was removed and 100 µL of DMSO was added in each well. Cell viability was measured at 490 nm by spectrophotometer and IC50 was determined by SPSS. The proliferation rates were calculated using the following equation: proliferation index (%)= [(OD drug–OD control)/OD control]×100%.

LX-2 cells proliferation rates were determined by MTT assay. LX-2 cells were treated with apelin-13 at concentrations of 0–0.5µM for 12, 24, 48 and 72 h.

**The detection of ERK signaling in HepG2 cells**

The detection of ERK pathway in apelin-treated hepG2 cells. HepG2 cells were treated with 100 nM apelin-13 for the indicated time points. Then, the cells were collected, and the total cell lysates were analyzed by Western blot using antibodies against p-ERK and ERK. β-actin was used as the endogenous control.
